# Distinct gut microbiota composition among older adults with myocardial ageing

**DOI:** 10.1002/ehf2.14139

**Published:** 2022-09-07

**Authors:** Jie Jun Wong, Rikky W. Purbojati, Ru‐San Tan, Sven Pettersson, Angela S. Koh

**Affiliations:** ^1^ National Heart Centre Singapore Singapore Singapore; ^2^ Singapore Center on Environmental Life Sciences Engineering (SCELSE) Nanyang Technological University Singapore Singapore; ^3^ Duke‐NUS Medical School Singapore Singapore; ^4^ Department of Neurobiology, Care and Society Karolinska Institute 171 77 Stockholm Sweden; ^5^ National Neuroscience Institute Tan Tock Sing Hospital Singapore 308433 Singapore; ^6^ Faculty of Medical Sciences Sunway University Kuala Lumpur 47500 Malaysia

Changes in cardiac structure and function occur with ageing and may lead towards ageing‐related cardiovascular disease.^1^ Recent explorations into intestinal microbiota have provided important insights into shifts in microbial composition that occur in response to cardiovascular disease pathogenesis. Several proposed mechanisms include altered gut permeability, endotoxemia, and the systemic effect of metabolites including trimethylamine (TMA), short‐chain fatty acids (SCFA), and secondary bile acids.^1^ However, causal associations between gut microbes and left ventricular (LV) function have yet to be proven. We sought to determine whether gut microbial composition is associated with left ventricular myocardial relaxation, an early manifestation of myocardial ageing, among older adults.

Among *n* = 15 participants (53% males, mean age 75 ± 4 years) recruited as part of a community‐based research study on myocardial ageing, subjects with normal LV ejection fraction (60% and above) on baseline echocardiography were selected to undergo gut microbial composition examination in this proof‐of‐concept cross‐sectional study. Myocardial ageing was assessed to be more impaired in those subjects with lower calculated ratios of peak early (E) to late diastolic (A, atrial contraction) velocities on Doppler echocardiography. Metagenomic reads from stool samples were processed after excluding human reference genome data and analysed using the lowest common ancestor algorithm into microbial taxonomical classifications. Statistical analysis was performed with linear discriminant analysis method. We compared metagenomic reads between older adults with myocardial ageing (*n* = 8) vs. those without myocardial ageing (*n* = 7), based on E/A ratios (E/A 0.77 vs. E/A 1.28, *P* < 0.0001). Older adults with greater myocardial ageing had a higher prevalence of *Bacteroidetes* phyla and *Bacteroidaceae* family, including *Bacteroides xylanisolvens* , *Bacteroides* sp. *2 1 22*, and *Bacteroides* sp. *4 1 36*. There was also a higher prevalence of *Paraprevotella* genus including *Paraprevotella xylaniphila*, as well as certain *Firmicutes* species including *Ruminococcus* sp. *CAG9*, *Clostridium* sp. *CAG791*, *Firmicutes bacterium* sp. *CAG227*, and *F. bacterium sp. CAG791*. The largest relative effect size, using logarithmic linear discriminant analysis (LDA; *Figure* 1), was with *Bacteroides* sp. including *B. xylanisolvens* and *Bacteroides* sp. *4 1 36* (log 3.0). In contrast, older adults without myocardial ageing had more bacterium from the *Firmicutes* phyla, namely, *Peptostreptococcaceae* family including *Clostridioides* genus and *Clostridioides difficile*, as well as *Clostridiaceae* family, *Oscillibacter* sp. *CAG155*, *F. bacterium* sp. *CAG212*, *F. bacterium sp. CAG341*, and *Blautia producta* . We also observed a higher prevalence of *Prevotella timonensis* from the *Bacteroidetes* phyla. The largest effect size was with *Oscillibacter* sp. *CAG155* (LDA log 2.8), followed by *F. bacterium sp. CAG212* and *Peptostreptococcaceae* family LDA (log 2.5).

**Figure 1:**
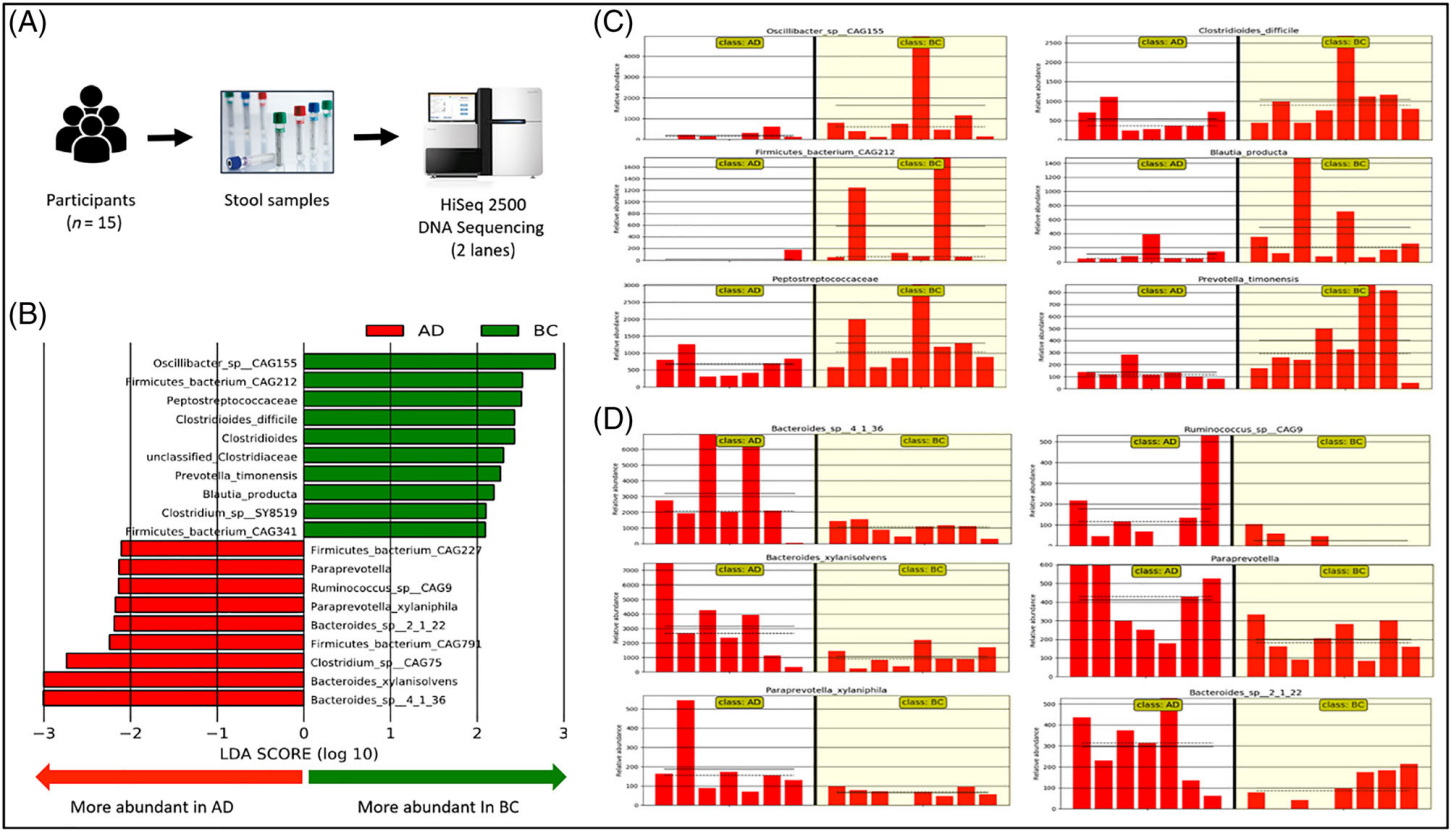
Distinct groups of gut microbiota exist between individuals with myocardial ageing (Group AD) and those without (Group BC). (A) DNA of stool samples from 15 subjects were extracted and sequenced on two lanes of HiSeq 2500 using Rapid‐Run Mode with 2 × 250 bp paired‐end reads length. After quality trim and filtering, the average sequence yield per subject is 1 797 835 paired‐end reads. (B) Linear discriminant analysis (LDA) of microbial taxa in patients with significant myocardial ageing vs. without those without myocardial ageing. The length of the bar corresponds to the LDA score that represents the effect size difference of a taxon abundance between one group and the other. Higher LDA scores represent larger effect size compared with the other cohort. (C and D) Taxa abundance plots show the top 6 bacteria species that are over‐abundant in the Group BC (C; without myocardial ageing) and Group AD (D; with myocardial ageing) as measured by LEFse (Kruskal–Wallis < 0.05, LDA score > 2). The solid horizontal line indicates the mean abundance level, and the dashed horizontal line indicates the median abundance level.

Older adults with cardiac ageing had higher levels of several pathogenic gut bacteria. *Ruminococcus*, of the phylum *Firmicutes*, have been associated with higher C‐reactive protein levels and higher pulse wave velocity.^1^ Certain *Ruminococcus* species are also capable of producing TMA, which has been linked to atherosclerotic disease and heart failure.^1^ Several *Paraprevotella* species, including *P. xylaniphila*, can produce pro‐inflammatory metabolites, such as succinic acid, and is also associated with hypertension, metabolic diseases, and inflammatory diseases.^2^ Increase in gut *Paraprevotella* has been observed in association with the development of heart failure in mice.^2^

Among individuals without cardiac ageing, we found higher levels of *Firmicutes* bacteria. *Firmicutes* are producers of SCFAs that regulate cholesterol levels, and some species have been associated with higher serum HDL.^1^ Reduced levels of *Firmicutes* were associated with LV hypertrophy and progression to heart failure in rats.^2^ In conjunction with higher levels of *Bacteroidetes* bacteria seen in our samples with cardiac ageing, we speculate that the balance between *Firmicutes* and *Bacteroidetes* (i.e., ratio) may be useful for studying gut microbial composition in relation to myocardial ageing in the future. The *Firmicutes* to *Bacteroidetes* ratio, a marker used for gut dysbiosis, was recently observed to be non‐significantly lower in patients with heart failure with preserved ejection fraction.^3^ Reductions in *Oscillibacter* have been associated with higher serum C‐reactive protein, progression to heart failure, and gut dysbiosis.^1^, ^2^

Nevertheless, gut microbiology is influenced by multiple other factors including comorbidities, medications, diet, and exercise; thus, larger cohorts and statistical matching are warranted to confirm these findings. Greater in‐depth speciation of the analysed samples may add precision in delineating different bacterial metabolic pathways and aid our understanding of the mechanisms behind the observed associations. Traditional biodiversity indices, such as alpha and beta diversity, would be necessary to investigate definitively for gut dysbiosis.^4^ Lastly, the cross‐sectional nature of our study prohibits causal inferences. However, existing literature suggest that core dominant bacterial species appear to be stable over time in the gut (in contrast to dynamic serum profiles).^5^ This stability emphasizes an opportunity for preliminary road maps into microbiome‐associated cardiac ageing.

In conclusion, myocardial ageing was associated with distinct groups of pathogenic gut microbiota in this proof‐of‐concept study. These findings are hypothesis‐generating and may be useful for future work in ageing‐related cardiovascular disease.

## Funding

This work was supported by the National Medical Research Council of Singapore (MOH‐000153; HLCA21Jan‐0052) and Hong Leong Foundation.
